# The G-protein-coupled bile acid receptor Gpbar1 (TGR5) suppresses gastric cancer cell proliferation and migration through antagonizing STAT3 signaling pathway

**DOI:** 10.18632/oncotarget.5353

**Published:** 2015-09-21

**Authors:** Cong Guo, Jia Su, Zhijun Li, Rui Xiao, Jianxun Wen, Yanyan Li, Meng Zhang, Xueting Zhang, Donna Yu, Wendong Huang, Wei-Dong Chen, Yan-Dong Wang

**Affiliations:** ^1^ State Key Laboratory of Chemical Resource Engineering, College of Life Science and Technology, Beijing University of Chemical Technology, Beijing, P. R. China; ^2^ Key Laboratory of Molecular Pathology, School of basic medical science, Inner Mongolia Medical University, Hohhot, Inner Mongolia, P. R. China; ^3^ Key Laboratory of Receptors-Mediated Gene Regulation and Drug Discovery, School of Medicine, Henan University, Kaifeng, Henan, P. R. China; ^4^ Department of Diabetes and Metabolic Diseases Research, Beckman Research Institute, City of Hope National Medical Center, Duarte, California, USA

**Keywords:** Gpbar1, TGR5, gastric cancer, STAT3, bile acid receptor

## Abstract

Gpbar1 (TGR5), a membrane-bound bile acid receptor, is well known for its roles in regulation of energy homeostasis and glucose metabolism. Here we show that TGR5 is a suppressor of gastric cancer cell proliferation and migration through antagonizing STAT3 signaling pathway. We firstly show that TGR5 activation greatly inhibited proliferation and migration of human gastric cancer cells and strongly induced gastric cancer cell apoptosis. We then found that TGR5 activation antagonized STAT3 signaling pathway through suppressing the phosphorylation of STAT3 and its transcription activity induced by lipopolysaccharide (LPS) or interleukin-6. TGR5 overexpression with ligand treatment inhibited gene expression mediated by STAT3. It suggests that TGR5 antagonizes gastric cancer proliferation and migration at least in part by inhibiting STAT3 signaling. These findings identify TGR5 as a suppressor of gastric cancer cell proliferation and migration that may serve as an attractive therapeutic tool for human gastric cancer.

## INTRODUCTION

Gastric cancer or stomach cancer, an inflammation-associated cancer, is the most common cause of cancer-related death in the world [1, 2]. Despite many years of extensive research, there is still no effective treatment and prognosis of gastric cancer is poor, which causes that gastric cancer represents the third leading cause of cancer mortality worldwide [3–6]. Understanding the mechanisms of gastric cancer and development of novel approaches to predict or treat gastric cancer are urgent for saving the lives of a large number of patients (723,000 deaths occurred worldwide in 2012, from http://www.who.int/mediacentre/factsheets/fs297/en/).

Signal transducer and activator of transcription 3 (STAT3) has received considerable attention as a key inhibitor of inflammation and cancer. STAT3 is a transcription factor, and belongs to STAT family [7]. It is activated in response to various cytokines and growth factors. STAT3 activation requires transient phosphorylation of cytoplasmic monomers that dimerize, translocate to the nucleus, and bind to specific DNA sequences [8]. Under normal conditions, STAT3 activation is transient and tightly controlled. Conversely, chronic activation of STAT3 signaling is frequently detected in numerous human inflammatory diseases and cancer, including gastric tumorigenesis [9, 10]. Mounting evidence supports the notion that constitutive STAT3 activation is fundamental to the pathobiology of these human diseases. Therefore, defining new therapeutic targets that antagonize STAT3 signaling is crucial for further understanding the regulation of this signaling pathway and the development of novel therapeutic strategies to inhibit prolonged activation of this pathway in human cancer.

The bile acid receptor TGR5 is a regulator of energy homeostasis, bile acid homeostasis as well as glucose metabolism [11]. TGR5 is a member of the G-protein-coupled receptor (GPCR) family which contains 7 transmembrane domains and transduces extracellular signals through heterotrimeric G proteins [12]. We and other group reported that TGR5 is a suppressor of NF-κB-mediated inflammation [13]. The notion that chronic inflammation is a frequent cause of cancer is well documented [3, 14]. Disrupting the aberrant activation of STAT3 or NF-κB signaling is able to dramatically suppress tumor progression [4]. Therefore, the previous results raise the possibility that TGR5 may be a suppressor of inflammation-related cancer such as gastric cancer.

In this study, we show that TGR5 activation inhibited proliferation and migration of human gastric cancer cells and induced gastric cancer cell apoptosis. Furthermore, it is found that TGR5 activation dramatically suppressed STAT3-midated target genes. We identify that TGR5 is a suppressor of STAT3 signaling pathways in gastric cancer cells via suppressing STAT3 phosphorylation and its transcription activity. These findings suggest TGR5 may be a potential target for therapeutic intervention in human gastric cancer through antagonizing STAT3 signaling.

## RESULTS

### TGR5 activation impairs proliferation, migration and invasion of human gastric cancer cells

**Figure 1:**
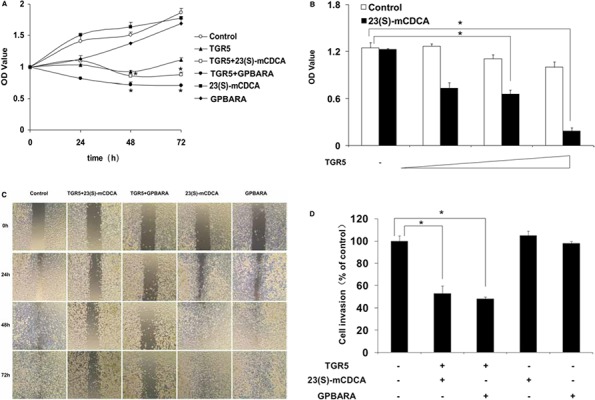
TGR5 activation impairs proliferation and migration of human gastric cancer cells **A.** TGR5 activation by its ligand inhibited proliferation of SGC7901 cells. Proliferation of cells was analyzed using MTT assay. TGR5 plasmid was transfected into SGC7901 cells and then the ligand was added into the culture. After 24, 48 and 72 hours of treatment, MTT assay was performed to determine cell proliferation. *P < 0.05 versus the control groups (*n* = 3). **B.** TGR5 activation by 23(S)-mCDCA inhibited proliferation of SGC7901 cells in a TGR5-dose dependent manner. SGC7901 cells were transfected with 80ng, 160ng and 320ng of TGR5 expression plasmid. Then after 24 hours of transfection, the ligand was added into the culture. After 48 hours of treatment, MTT assay was performed to determine cell proliferation. *P < 0.05 (*n* = 3). **C.** TGR5-transfected cells with ligand treatment exhibited a lower scratch closure rate than the controls in *in vitro* scratch assay (*n* = 3). The experiments were performed in triplicate and a representative of three independent experiments was shown. **D.** *In vitro* cell invasion assay shown that TGR5 activation inhibited SGC7901 cell invasion (*n* = 3). *P < 0.05 versus the control groups.

It has been well known that the potential of cells to migrate, to grow invasively, or to proliferate is the most important cancer-causing factor. To determine how TGR5 affected gastric tumor growth and progression, we transfected TGR5 overexpression plasmid to SGC7901 gastric cancer cells and examined the effect of TGR5 activation by its ligand, 23(S)-mCDCA and 3-(2-Chlorophenyl)-N-(4-chlorophenyl)-N,5-dimethylisoxazole -4-carboxamide (GPBARA), on SGC7901 cell proliferation, migration and invasion. As shown in MTT results, GPBARA treatment suppresses the growth of SGC7901 cells. TGR5 overexpression enhanced this suppression (Fig. 1A). Although 23(S)-mCDCA treatment did not affect cell growth, 23(S)-mCDCA with TGR5 overexpression obviously suppressed the growth of SGC7901 cells (Fig. 1A). Transfection of these cells with TGR5 inhibited cell growth in the absence of ligand, suggesting that TGR5 may suppress cell growth without the addition of exogenous ligand, possibly resulting from the fact that GPCRs have constitutive activity as previously reported [13, 15]. Furthermore, it was found that TGR5 activation by 23(S)-mCDCA suppressed cell proliferation in a TGR5-dose dependent manner (Fig. 1B). Meanwhile, *in vitro* scratch assay was performed to test human gastric cancer cell migration. TGR5-transfected cells with ligand treatment exhibited a lower scratch closure rate than the controls (Fig. 1C). *In vitro* cell invasion assay was also performed. We found that TGR5-transfected cells with ligand treatment displayed lower invasion compared with the control group (Fig. 1D). These results showed that TGR5 activation impaired proliferation, migration and invasion of human gastric cancer cells, which may contribute to suppress gastric cancer development.

### TGR5 activation induced apoptosis of human gastric cancer cells

**Figure 2:**
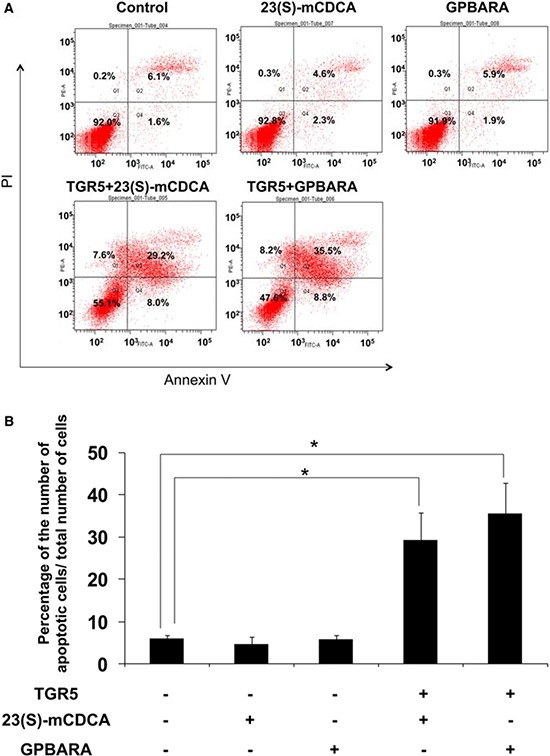
TGR5 activation induced apoptosis of SGC7901 cancer cells **A.** The figure shows representative fluorescence-activated cell sorting analysis of SGC7901 cells. Cells were transfected with TGR5 expression plasmid and then treated with the ligands for 48 hours. Cells were stained with BD Pharmingen Annexin V: FITC Apoptosis Detection Kit I for Flow cytometric analysis. The experiments were performed in triplicate and a representative of three independent experiments was shown. **B.** SGC7901 cells were treated as described above. Apoptotic cells were counted as a percentage of the total number of cells using fluorescence-activated cell sorting. Data are the means of three independent experiments ± SD. *P < 0.05 versus the control groups.

Next we test whether TGR5 activation induced gastric cancer cell apoptosis. The apoptotic effects of TGR5 activation were tested using Annexin V-FITC and Propidium Iodide (PI) apoptosis double staining. The results show that, compared with the control group, GPBARA or 23(S)-mCDCA treatment without TGR5 overexpression did not induce gastric cancer cell apoptosis. TGR5 overexpression with ligand treatment caused obviously apoptotic cell increase (The apoptotic cell percentages increased from 6.1 ± 0.5% in the control group to 35.5 ± 2.5% and 29.2 ± 3.1% for GPBARA and 23(S)-mCDCA treatment, respectively) (Fig. 2). These results suggest that TGR5 activation induced gastric cancer cell apoptosis.

### TGR5 Inhibits phosphorylation of STAT3

**Figure 3:**
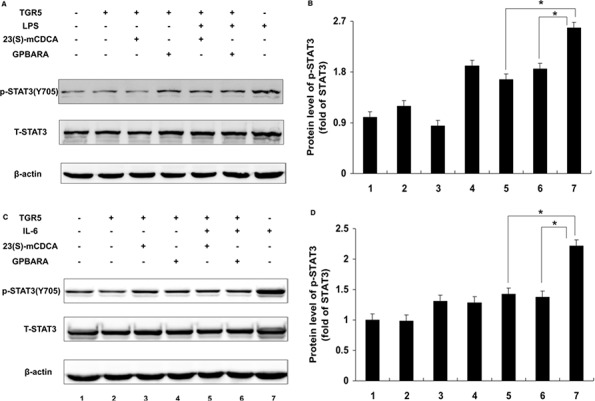
TGR5 inhibits STAT3 phosphorylation at Tyr705 **A.** TGR5 overexpression with ligand treatment suppressed LPS-induced phosphorylation of STAT3 at Tyr705 in SGC7901 cells. Cells were treated with ligand for 24 hours and then were treated with LPS for 6 hours. (*n* = 3) p-STAT3(Y705), phosphorylated STAT3 at Tyr705; T-STAT3, total STAT3. β-actin as a loading control. **B.** The data of relative protein levels in A. are expressed as fold change over the ratio of p-STAT3(Y705) to T-STAT3 in the control group (lane 1). **C.** TGR5 overexpression with ligand treatment suppressed IL-6-induced p-STAT3 at Tyr705 in SGC7901 cells. Cells were treated with ligand for 24 hours and then were treated with IL-6 for 6 hours. (*n* = 3) p-STAT3(Y705), phosphorylated STAT3 at Tyr705; T-STAT3, total STAT3. β-actin as a loading control. **D.** The data of relative protein levels in **C.** are expressed as fold change over the ratio of p-STAT3(Y705) to T-STAT3 in the control group (lane 1). *P < 0.05.

Constitutive activation of IL-6/STAT3 signaling has been detected in a wide variety of human cancers and is considered as an important factor for cancer initiation, development, and progression [7, 9]. The previous reports show that direct STAT3 suppression induced cancer cell apoptosis [16, 17]. If TGR5 is a suppressor of STAT3, TGR5 activation may inhibit STAT3 phosphorylation. We tested the suppression of TGR5 activation on phosphorylation of STAT3 at Tyr705 and Ser727. Compared with the control group, lipopolysaccharide (LPS) induced phosphorylation of STAT3 at both Tyr705 and Ser727 (Fig. 3A, 3B and Supplementary Fig. S1A, B) in SGC7901 cancer cells. TGR5-transfected SGC7901 cells with ligand treatment (23(S)-mCDCA and GPBARA) inhibited LPS-induced STAT3 phosphorylation at Tyr705 by about 36% and 28%, respectively (Fig. 3A, 3B), but not at Ser727 (Supplementary Fig. S1A, 1B). Furthermore, we used interleukin-6 (IL-6) induction to confirm this result. IL-6 induced phosphorylation of STAT3 at both Tyr705 and Ser727 (Fig. 3C, 3D and Supplementary Fig. S1C, D). TGR5 activation by both 23(S)-mCDCA and GPBARA suppresses IL-6-induced phosphorylation of STAT3 at Tyr705 by about 36% and 38%, respectively (Fig. 3C, 3D), but not at Ser727 (Supplementary Fig. S1C, 1D). These results demonstrated that TGR5 activation is able to suppress STAT3 phosphorylation at Tyr705. In this study, the test of inhibition of STAT3 using its inhibitor S3I-201, as a positive control, has been performed (Supplementary Fig. S2).

### TGR5 activation suppresses STAT3 target gene expression

**Figure 4:**
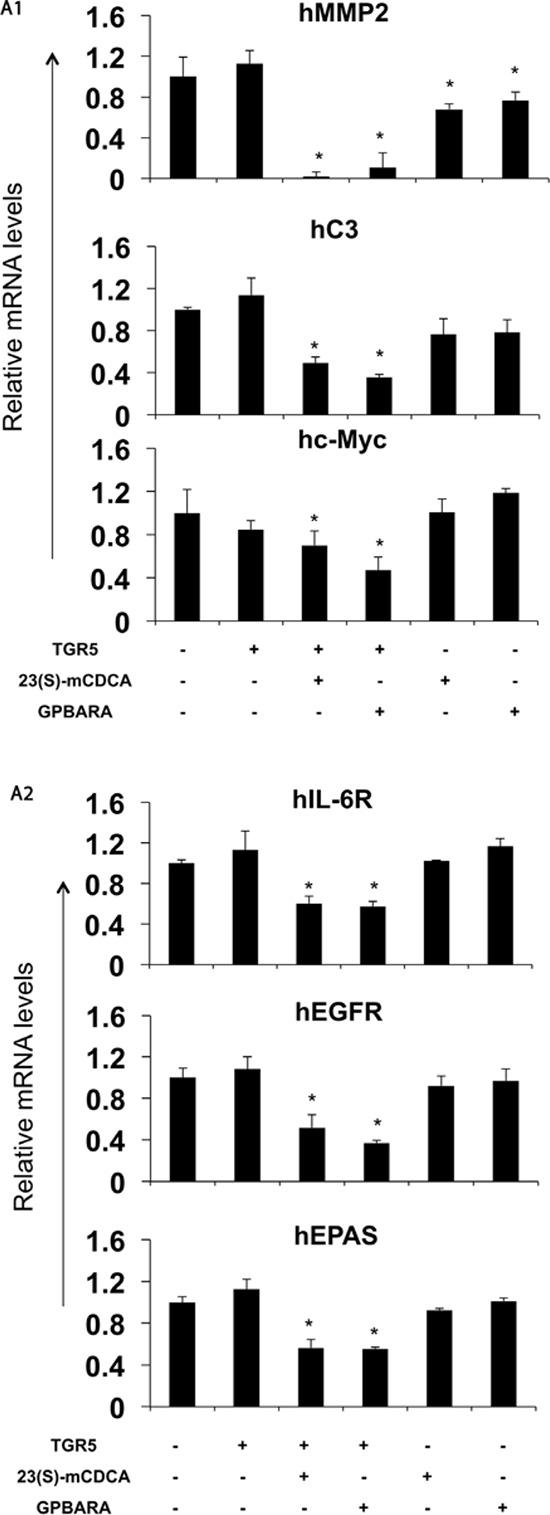

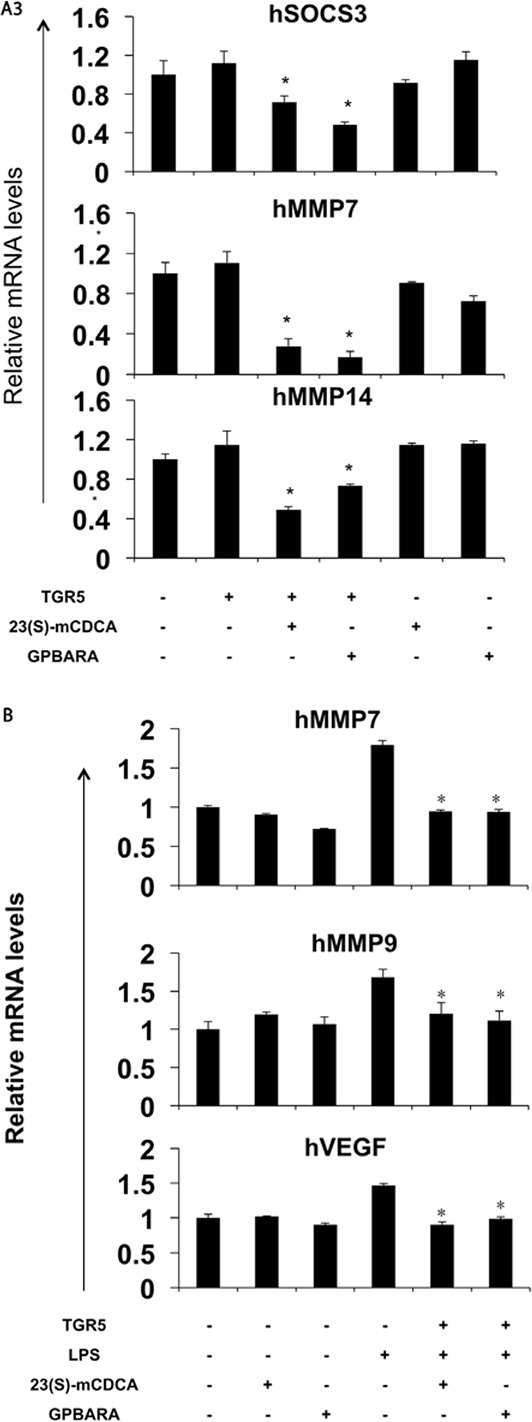
TGR5 activation suppresses STAT3 target gene expression **A.** TGR5 ligand treatment suppresses MMP2 and C3 gene expression. 23(S)-mCDCA and GPBARA treated SGC7901 cells for 24 hours. TGR5 overexpression with ligand treatment suppresses MMP2, C3, c-Myc, IL-6R, EGFR, EPAS, SOCS3, MMP7 and MMP14 gene expression. SGC7901 cells were transfected with the TGR5 expression plasmid or control plasmid. After transfection, cells were treated with GPBARA (3 μM), 23(S)-mCDCA (10 μM) or vehicle (DMSO) for 24 hours. *P < 0.05 versus the control group (without any treatment). (*n* = 3). **B.** TGR5 activation suppresses LPS-induced gene expression. SGC7901 cells were transfected with the TGR5 expression plasmid or control plasmid. After transfection, cells were treated with GPBARA (3 μM), 23(S)-mCDCA (10 μM) or vehicle (DMSO) for 24 hours. Then cells were treated with LPS for 6 hours. *P < 0.05 versus the LPS-treated group. (*n* = 3).

Next, We tested whether TGR5 activation could inhibit STAT3-mediated gene expression. In SGC7901 gastric cancer cells, TGR5 overexpression alone did not affect the expression of most genes tested. TGR5 ligands 23(S)-mCDCA and GPBARA suppresses gene expression of matrix metalloproteinases (MMP) 2 and complement component 3 (C3) mediated by STAT3 (Fig. 4A). TGR5 overexpression with the ligands 23(S)-mCDCA or GPBARA treatment repressed gene expression of MMP2, C3, c-Myc, interleukin 6 receptor (IL-6R), epidermal growth factor receptor (EGFR), endothelial PAS domain protein 1 (EPAS), suppressor of cytokine signaling 3 (SOCS3), MMP7 and MMP14 mediated by STAT3 (Fig. 4A). Furthermore, TGR5 activation suppressed LPS-induced MMP7, MMP9 and vascular endothelial growth factor (VEGF) expression (Fig. 4B). Together, these results show that TGR5 activation suppressed STAT3-mediated gene expression

### Activation of TGR5 antagonizes STAT3 signaling in gastric cancer cells

**Figure 5:**
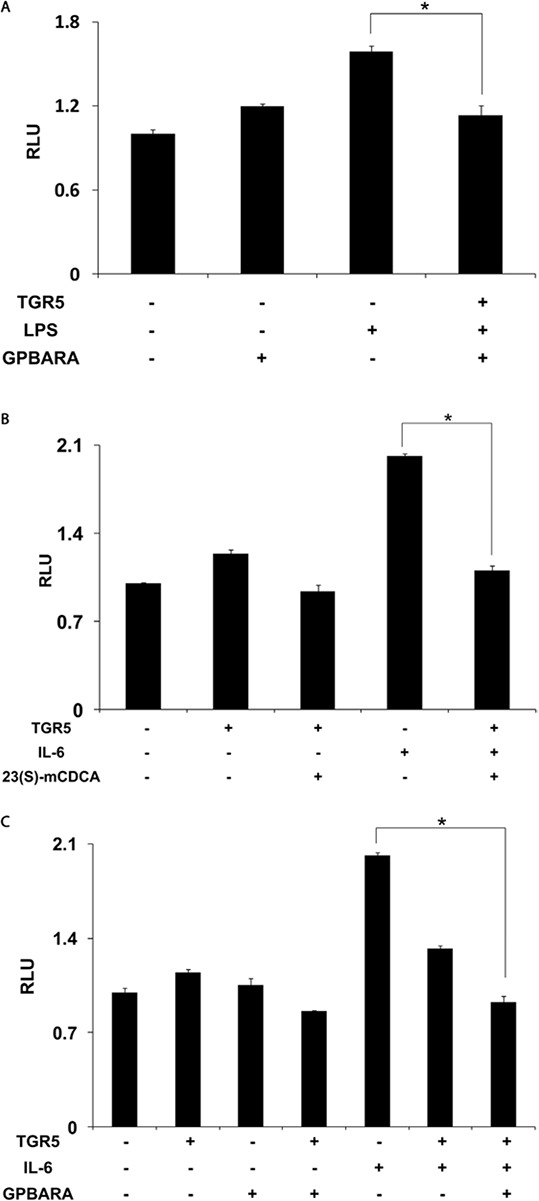
Activation of TGR5 antagonizes STAT3 transactivity **A.** TGR5 suppressed STAT3 transactivity induced by LPS. SGC7901 cells were cotransfected with the STAT3 reporter plasmid (pSTAT3-LUC), phRL-TK, and TGR5 expression plasmid. After transfection, cells were treated with GPBARA (3 μM) or vehicle (DMSO) for 24 hours and then treated with LPS (40 μg/mL) for 6 hours. **B.** TGR5 ligand 23(S)-mCDCA suppressed STAT3 transactivity induced by IL-6 (50 ng/mL). **C.** TGR5 ligand GPBARA suppressed STAT3 transactivity induced by IL-6 (50 ng/mL). *P < 0.05 versus the LPS or IL-6-treated groups. RLU, relative luciferase units. (*n* = 3).

Because TGR5 activation by 23(S)-mCDCA and GPBARA inhibited the expression of STAT3 target genes, we next tested whether TGR5 activation inhibited STAT3 activity at the level of gene transcription. We cotransfected SGC7901 cells with a STAT3 reporter plasmid and the control plasmid phRL-TK and assessed the effects of TGR5 activation on the regulation of STAT3 reporter activity. Treatment with LPS resulted in about 1.6-fold greater STAT3 reporter activity. STAT3 activity induced by LPS was inhibited by GPBARA treatment with TGR5 overexpression (Fig. 5A). Treatment with a known STAT3 pathway activator IL-6 resulted in 2.0-fold greater STAT3 reporter activity (Fig. 5B, 5C). TGR5 overexpression with 23(S)-mCDCA or GPBARA represses IL-6-induced STAT3 reporter activity by about 45% (Fig. 5B) and 54% (Fig. 5C), respectively. These results indicate that activation of TGR5 can antagonize STAT3 activity at the level of gene transcription in gastric cancer cells.

## DISCUSSION

The antitumor activities of proteins include the inhibition of proliferation, growth arrest in the cell cycle, enhanced apoptosis and the modulation of signaling pathways. Our previous report show that TGR5 activation antagonizes NF-κB-mediated liver inflammation [13]. In the present work, we demonstrate that TGR5 activation reduces SGC7901 gastric cancer cell proliferation and migration and enhances gastric cancer cell apoptosis. Furthermore, it is found that TGR5 activation antagonizes STAT3 signaling in gastric cancer cells through inhibiting STAT3 transcriptional activity and phosphorylation. These results suggest that TGR5 is a suppressor of gastric cancer through antagonizing STAT3 signaling.

TGR5 belongs to GPCR family [13]. GPCRs comprise the largest protein family of transmembrane receptors that sense molecules outside the cell and activate inside signal transduction pathways through agonist binding to an orthosteric binding site. GPCRs regulate cell migration, proliferation, differentiation and survival and play a major role in the development and progression of many diseases such as inflammatory diseases and cancer [18]. Many GPCRs contribute to tumor cell growth [19], while only a few of GPCRs suppress cancer development [20]. For example, GPR43 activation suppresses colon cancer by suppressing cell proliferation and inducing apoptotic cell death [20]. Here, our results show that TGR5 is a potential gastric tumor suppressor.

STAT3, a transcription factor that can promote oncogenesis, is commonly activated in cancer [21]. The constitutive activation of STAT3 is frequently detected in clinical samples from a wide range of human carcinoma such as multiple myeloma, glioblastoma, colorectal and hepatocellular carcinoma [22, 23]. Importantly, elevated levels of STAT3 phosphorylation were correlated with tumor invasion, metastasis, and worse prognosis in colorectal, hepatocellular and other carcinoma [23, 24]. Blocking constitutive STAT3 signaling in carcinoma cells by STAT3 antisense oligonucleotides, STAT3 small interfering RNAs (siRNAs), or stable transfection of dominant-negative STAT3 can inhibit cancer cell growth, invasion and metastasis, and induce apoptosis [22]. Moreover, inhibition of constitutive STAT3 signaling by the JAK2 inhibitor AG490 [25] suppressed the growth and invasion of human hepatocellular carcinoma cells, and also induced apoptosis in multiple myeloma cells [26]. These findings suggest that constitutive STAT3 signaling is crucial to the survival, invasion, and growth of human carcinoma cells. Targeting the STAT3 pathway directly could be a promising and novel form of treatment for these human cancers. In this study, our data show that TGR5 activation strongly suppresses STAT3 signaling by antagonizing STAT3 phosphorylation and its transactivity, which raises the high possibility that TGR5 is a suppressor of STAT3 signaling pathway. It would be interesting to further study the molecular mechanisms by which TGR5 activation suppresses these steps in STAT3 signaling pathway.

It is known that the matrix metalloproteinases are important regulators in cancer cell growth, invasion and migration [27, 28]. In the current work, we found that TGR5 activation inhibited MMP2, MMP7 and MMP14 gene expression in gastric cancer cells. Furthermore, we noted that TGR5 activation suppressed gastric cancer cell proliferation, migration and invasion. These results indicate that TGR5 may regulate MMPs to suppress cancer cell migration. One of mechanisms by which TGR5 activation inhibits MMPs may be through antagonizing STAT3 signaling pathways because these MMPs are the target genes of STAT3 [29, 30].

It has been reported that TGR5 could be a potential target for the treatment of diabesity and associated metabolic disorders [31, 32]. For example, Watanabe et al. reported that TGR5 activation by bile acids induces energy expenditure in muscle and brown adipose tissue [31]. Thomas et al. found that TGR5 activation improves glucose tolerance and insulin sensitivity in fat-fed mice [32]. These diseases, such as obesity, insulin resistance and type 2 diabetes, are also closely associated with chronic inflammation characterized by abnormal cytokine production, increased acute-phase reactants, and activation of a network of inflammatory signaling pathways [33]. Combining with our previous study [13], our results show that TGR5 is a suppressor of gastric carcinogenesis probably by antagonizing both NF-κB and STAT3 pathways. Therefore, there is a potential link between anti-cancer and treatment of obesity and diabetes through TGR5. TGR5 may be an attractive therapeutic target not only for metabolic disorders but also for cancer.

In conclusion, our results reveal that TGR5 is a suppressor of gastric cancer cell proliferation and migration and TGR5 activation suppresses STAT3 signaling pathway, indicating that TGR5 ligands have utility in anti-gastric cancer. These findings suggest TGR5 is a potential target for anti-cancer drug design and its agonist ligands offer possible therapies to prevent and treat gastric cancer.

## MATERIALS AND METHODS

### Reagents and plasmids

LPS (from Escbricbia coli 0111:B4) was purchased from Sigma Chemical (St Louis, MO). IL-6 was purchased from PeproTech. 23(S)-mCDCA was provided by Dr. Wendong Huang and Dr. Donna Yu (City of Hope, Duarte, CA). GPBARA (TGR5 Receptor Agonist, 3-(2-Chlorophenyl)-N-(4-chlorophenyl)-N,5 -dimethylisoxazole-4-carboxamide) was purchased from BioVision (Milpitas, CA). The pmTGR5 and pSTAT3-LUC expression vectors were created in our laboratory. The mouse TGR5 gene was cloned into pIRESneo3 (Clontech) plasmid to generate pmTGR5 overexpression plasmid. The minimal TA promoter (23bp) was used for pSTAT3-LUC vector. The phRL-TK vector was kindly provided by Akio Kruoda (City of Hope, Duarte, CA).

### Cell culture and transfection

Gastric cancer cell line SGC7901 was obtained from Institute of Basic Medical Sciences (IBMS) of Chinese Academy of Medical Sciences. Cells were grown in complete culture medium (RPMI-1640 [with L-glutamihe] supplied with 10% (vol/vol) inactivated fetal calf serum and 1% (vol/vol) antibiotics-antimycotics). Cultures were fed with fresh medium twice weekly. For experiments, 8 × 10^5^ SGC7901 cells were seeded in 60 mm culture dishes with complete culture medium. Transient transfection of SGC7901 cells with TGR5 expression plasmid was performed using Lipofectamine 2000 (Invitrogen, Carlsbad, CA). Twenty-four hours after transfection, cells were pre-treated with 23(S)-mCDCA (10 μM) or GPBARA (3 μM) for one day. Then cells were treated with or without LPS. Following a 6-hour incubation, cells were harvested for Quantitative Real-Time PCR analysis. For protein assay, cells were pre-treated with 23(S)-mCDCA (10 μM) or GPBARA (3 μM) for one day. Then cells were treated with LPS or IL-6 for the indicated times. Finally, cells were collected for total protein isolation and Western blot assay. For luciferase assay, transient transfection of SGC7901 cells with the STAT3 reporter plasmid, phRL-TK, and/or TGR5 expression plasmid was performed using Lipofectamine 2000 (Invitrogen, Carlsbad, CA). Twenty-four hours after transfection, cells were pre-treated with 23(S)-mCDCA (10 μM), GPBARA (3 μM) or vehicle (dimethyl sulfoxide (DMSO)) for 24 hours. Then cells were treated with/without IL-6 (50 ng/mL). After 6 hours of incubation, cells were harvested and the luciferase activity was determined using a dual-luciferase reporter assay system in accordance with the manufacturer's instructions (Promega, Madison, WI). Luciferase activities were normalized by co-transfection of the control thymidine kinase-driven Renilla luciferase plasmid, phRL-TK. Data are expressed as relative fold activation to that of non-stimulated (−) sets.

### RNA isolation and quantitative real-time polymerase chain reaction

Total RNA was extracted from SGC7901 cells using Tri-Reagent (Molecular Research Center, Inc., Cincinnati, OH). Quantitative real-time PCR was performed using the Power SYBR Green PCR Master Mix protocol (Applied Biosystems, Foster City, CA). Amplification of β-actin was used as an internal reference. β-actin primers were obtained from Ambion, Inc. (Austin, TX). Quantitative PCR analysis was conducted using the ABI 7500 Sequence Detection System. Primers sequences are available on request.

### Immunoblot analysis

At indicated time points after treatment, SGC7901 cells were lysed for 30 minutes with lysis buffer and centrifuged at 12,000 × g at 4°C for 15 minutes. The samples were resolved by 10% sodium dodecyl sulfate-polyacrylamide gel electrophoresis, transferred to nitrocellulose membranes, and blotted using primary antibodies (Cell Signaling Technology). The membranes were washed with Tris Buffered Saline with 0.1% Tween^®^ 20 (TBST) and then incubated with anti-rabbit secondary antibody conjugated to horseradish peroxidase (HRP) (1:5000) (Thermo Scientific, Waltham, MA). Bands on blots were visualized using Tanon 5200 enhanced chemiluminescence (ECL) detection system (Tanon, China) and quantified with a computerized digital imaging system using Tanon software.

### Cell proliferation assay

Cell proliferation was measured using the MTT assay every 24 hours. Briefly, 100 μL of cell suspension (5 × 10^4^/mL) was added to each well of a 96-well plate and incubated at 37°C for 24 hours. Then TGR5 plasmid was transfected to cells. After 24 hours, cells were treated with 10 μM of 23(S)-mCDCA or 3 μM of GPBARA. After 24, 48, or 72 hours of treatment, MTT reagent was added into cells. After 4 hours of incubation, 150 μL of dimethyl sulfoxide was added to dissolve formazancrystals, and optical density was measured at 570 nm.

### Flow cytometric assay for detection of apoptotic cell

After treatment with indicated reagents, suspended and attached SGC7901 cells were harvested by trypsinization and washed twice with PBS. Cells were stained with PI (20 μg/ml in PBS) and/or with Annexin V according to the manufacture's instructions (BD Pharmingen Annexin V: FITC Apoptosis Detection Kit I). Flow cytometric analysis was performed with a CyAn-ADP flow cytometer (DakoCytomation, Fort Collins, CO).

### *In vitro* scratch assay

For detection of cell migration by *in vitro* scratch assay, SGC7901 cells were cultured to confluent monolayers and then were transfected with/without TGR5 overexpression plasmid. Twenty-four hours after transfection, cells were treated with 23(S)-mCDCA (10 μM), GPBARA (3 μM) or vehicle (DMSO) for 24 hours, and then wounded by removing a 300–500 μm-wide strip of cells across the well with a standard 200 μL pipette tip. Wounded monolayers were washed twice to remove non-adherent cells. After indicated times of incubation, wound healing was recorded under a light microscopy.

### Cell invasion assay

Cell invasion was determined with QCM™ Cell Invasion Assay kit (Millipore) according to the manufacturer's instructions. SGC7901 cells were transfected with the TGR5 overexpression plasmid or control plasmid. Cells were cultured for 18 hours and then were collected for invasion assays. Cells were plated in the top chamber with polymerized collagen-coated membrane (24-well insert; pore size, 8 μm; Chemicon ECM551) with or without TGR5 ligand. The complete medium was placed in the lower chamber. After incubation at 37°C, cells that had invaded the lower surface of the membrane were extracted and cell counting was done with a standard microplate reader (at 560 nm). Data is represented as percent invasion based on the control group.

### Statistics

All data represent at least three independent experiments and are expressed as the mean ± SD. The two-way analysis of variance (ANOVA), followed by Bonferroni's post-hoc test, was performed. A *P* value less than 0.05 was considered significant.

## SUPPLEMENTARY FIGURES


