# Supplementary material for: The G-protein-coupled bile acid receptor Gpbar1 (TGR5) suppresses gastric cancer cell proliferation and migration through antagonizing STAT3 signaling pathway
Source: Oncotarget. 2015 Sep 21;6(33):34402–13. doi: 10.18632/oncotarget.5353 (PMC4741461; doi:10.18632/oncotarget.5353)
Supplement: Supplementary file 1 [file oncotarget-06-34402-s001.pdf]

SUPPLEMENTARY FIGURES

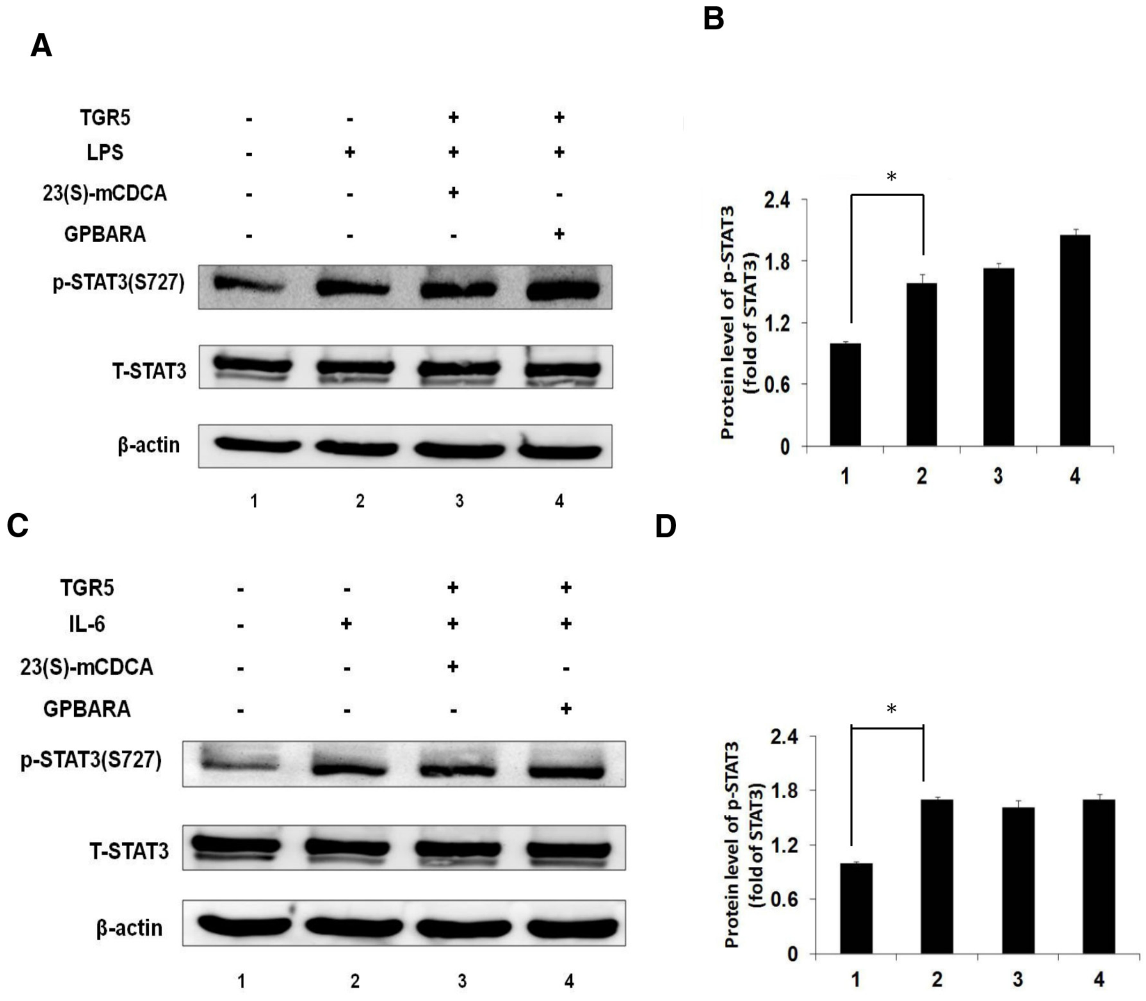

Supplementary Figure S1: TGR5 activation did not affect STAT3 phosphorylation at Ser727. A. TGR5 overexpression with ligand treatment did not affect LPS-induced phosphorylation of STAT3 at Ser727 in SGC7901 cells. Cells were treated with ligand for 24 hours and then were treated with LPS for 6 hours. ($n = 3$) p-STAT3(S727), phosphorylated STAT3 at Ser727; T-STAT3, total STAT3. β-actin as a loading control. **B.** The data of relative protein levels in (A) are expressed as fold change over the ratio of p-STAT3(S727) to T-STAT3 in the control group (lane 1). **C.** TGR5 overexpression with ligand treatment did not affect IL-6-induced phosphorylation of STAT3 at Ser727 in SGC7901 cells. Cells were treated with ligand for 24 hours and then were treated with IL-6 for 6 hours. ($n = 3$) **D.** The data of relative protein levels in (C) are expressed as fold change over the ratio of p-STAT3(S727) to T-STAT3 in the control group (lane 1). $^{*}P < 0.05$.

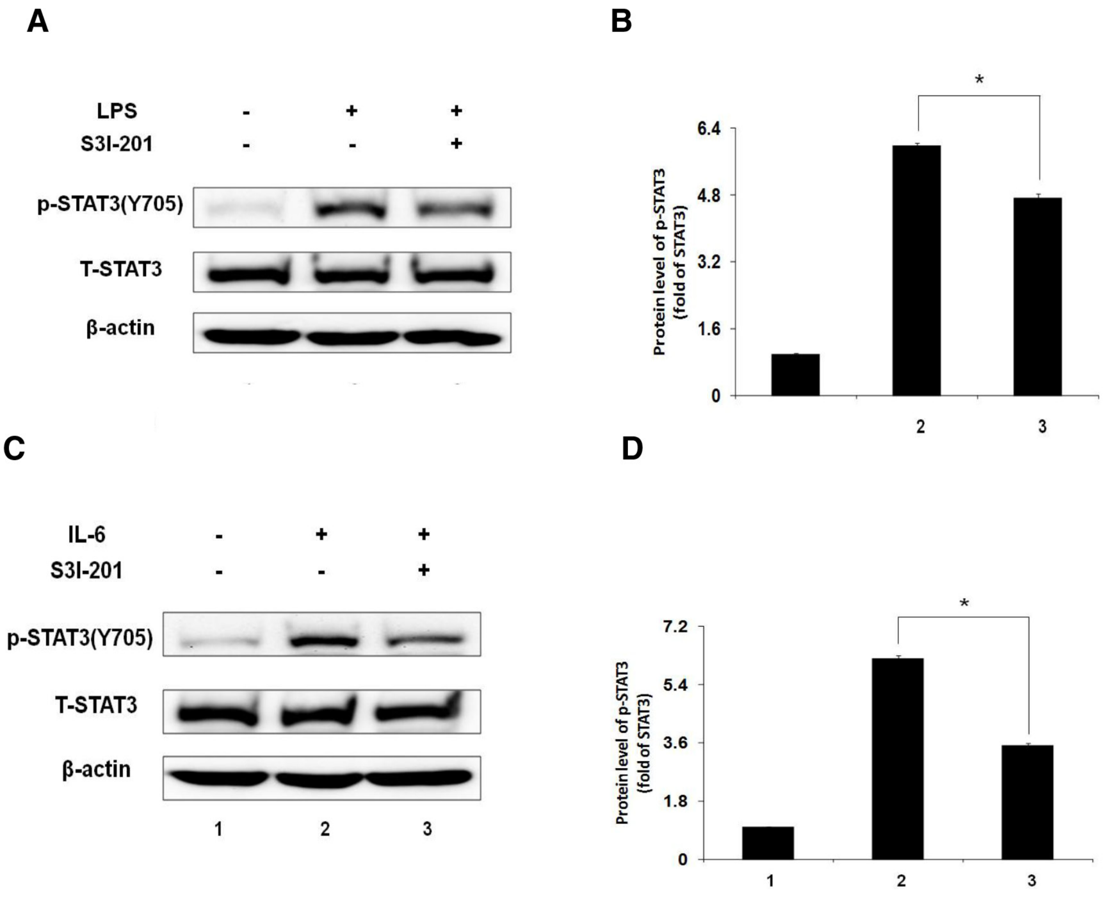

Supplementary Figure S2: The STAT3 inhibitor S3I-201 suppressed STAT3 phosphorylation at Tyr705 induced by LPS and IL-6. A. The inhibitor S3I-201 suppressed STAT3 phosphorylation at Tyr705 induced by LPS in SGC7901 cells. Cells were treated with the inhibitor S3I-201 (100μM) for 42 hours and then were treated with LPS for 6 hours. (n = 3) p-STAT3(Y705), phosphorylated STAT3 at Tyr705; T-STAT3, total STAT3. β-actin as a loading control. **B.** The data of relative protein levels in (A) are expressed as fold change over the ratio of p-STAT3 to T-STAT3 in the control group (lane 1). **C.** The inhibitor S3I-201 suppressed STAT3 phosphorylation at Tyr705 induced by IL-6 in SGC7901 cells. Cells were treated with the inhibitor S3I-201 (100μM) for 42 hours and then were treated with IL-6 for 6 hours. (n = 3) p-STAT3(Y705), phosphorylated STAT3 at Tyr705; T-STAT3, total STAT3. β-actin as a loading control. **D.** The data of relative protein levels in (C) are expressed as fold change over the ratio of p-STAT3 to T-STAT3 in the control group (lane 1). *$P < 0.05$.
